# Opinion: the evolving understanding of polygenic common variable immunodeficiency-like disorders

**DOI:** 10.3389/fimmu.2025.1618482

**Published:** 2025-08-28

**Authors:** Rohan Ameratunga, Euphemia Yee Leung, Hilary J. Longhurst, Klaus Lehnert, See-Tarn Woon

**Affiliations:** ^1^ Department of Clinical Immunology, Auckland Hospital, Auckland, New Zealand; ^2^ Department of Virology and Immunology, Auckland Hospital, Auckland, New Zealand; ^3^ Department of Molecular Medicine and Pathology, School of Medicine, Faculty of Medical and Health Sciences, University of Auckland, Auckland, New Zealand; ^4^ Maurice Wilkins Center, School of Biological Sciences, University of Auckland, Auckland, New Zealand; ^5^ Auckland Cancer Society Research Centre, School of Medicine, Faculty of Medical and Health Sciences, University of Auckland, Auckland, New Zealand; ^6^ Department of Medicine, School of Medicine, Faculty of Medical and Health Sciences, University of Auckland, Auckland, New Zealand; ^7^ Applied Translational Genetics, School of Biological Sciences, University of Auckland, Auckland, New Zealand

**Keywords:** CVID, polygenic, hypogammaglobinemia, TCF3, CGCDS

## Introduction

Common Variable Immunodeficiency Disorders (CVID) are the most frequent symptomatic Primary Immunodeficiency Disorder (PID) in both adults and children (1). Patients with CVID present with late-onset antibody failure with variable degrees of cellular immune dysfunction (2). Most patients with CVID experience recurrent and severe bacterial infections, but also have a predisposition to autoimmunity and inflammatory disorders.

The clinical manifestations of CVID can vary over time. Patients can initially experience recurrent infections in childhood but suffer autoimmune or inflammatory disorders later in life. Conversely, some patients with CVID present with autoimmunity and the diagnosis is made when immunoglobulins are measured before immunosuppression. In other CVID patients where immunoglobulins are not measured before treatment, severe infections can be precipitated by immunosuppression, unmasking the disorder.

## Genetics of CVID and CVID-like disorders

By definition, the precise genetic cause of CVID is unknown. In approximately 25% of non-consanguineous individuals, a causative autosomal dominant mutation underlies the PID (3). Families with autosomal dominant disorders frequently have variable penetrance and expressivity. In consanguineous societies, the majority of patients with primary antibody deficiencies have an underlying genetic defect, usually inherited as an autosomal recessive disorder. These patients typically present with severe disease early in life (4).

All current definitions of CVID exclude those with an underlying condition, which includes patients with causative mutations (5–7). These patients are deemed to have CVID-like disorders caused by a monogenic Inborn Error of Immunity (IEI) (8).

CVID-like disorders are characterized by marked locus heterogeneity (genocopy). Mutations in a large number of genes can result in a remarkably similar phenotype of impaired antibody production with variable degrees of cellular immune dysfunction (9). With the advent of Next Generation Sequencing (NGS) and more recently by second and third generation genome sequencing, it has become feasible to investigate disorders with marked locus heterogeneity.

In addition to causative mutations leading to an IEI, patients with CVID frequently have genetic variants which appear to predispose to, or enhance disease severity. The evidence these risk alleles do not cause CVID is based on several observations. First, the population prevalence of these alleles far exceeds that of CVID. The American College of Medical Genetics (ACMG) has published helpful information on the interpretations of genetic variants to determine if these are pathogenic or benign (10). The first consideration in the ACMG criteria is the frequency of the variant in the population: If it far exceeds the disease prevalence, it is unlikely to be causative.

Second, these variants do not segregate with disease in family studies (11). The ACMG criteria place considerable emphasis on family segregation studies. If these variants do not segregate with extended family studies, this is strong evidence they cannot be causative. It is important to note that CVID and CVID-like disorders can present later in life and these family segregation studies should include older family members as well as children. Large multi-generational kindreds where variants do not segregate with disease is strong evidence these are not pathogenic.

Last, the epistatic role of these variants was directly shown in a family carrying mutations of both *TNFSF13B/*TACI (C104R, c.310TC) variant and a nonsense mutation of *TCF3* (T168fsX191) (12). The proband who had both mutations was severely affected clinically compared to other members of the same family. She had a much higher CVID Disease Severity Score (CDSS) (13). Laboratory studies reflected these clinical observations, as the digenic proband had much lower *in vitro* antibody production, compared to other members of the kindred bearing only one mutation.

## Polygenic CVID-like disorders

The current Expert Committee on PIDs has deemed these disorders to be monogenic IEIs, although the majority of patients with PIDs do not have a causative pathogenic mutation (14). The genetic basis for CVID (by definition), selective IgA deficiency and Transient Hypogammaglobulinemia of Infancy (THI) are unknown. These three conditions numerically comprise by far the majority of PIDs.

The current Expert Committee on PIDs does not recognize polygenic causes of PIDs (8). With the advent of second and third generation sequencing, it has become increasingly obvious that many patients with CVID-like disorders have more than one mutation, which may be contributing to their phenotype.

Polygenic PIDs could influence the phenotype of an individual in several ways. If there is an epistatic effect between two genetic loci, there may be synergistic, non-linear interactions between two or more genes leading to a much more severe or much milder phenotype. Positive epistasis occurs where the digenic phenotype is much worse than in individuals bearing a single mutation. In contrast, negative epistasis occurs, where the deleterious effects of the two or more mutations are mitigated by one or both variants.

Quantitative epistasis typically occurs when gene products lie on the same signaling pathway. One gene mutation usually has a greater impact on the phenotype than the other. This is known as the epistatic hub and is often a gene product with non-redundant function such as a receptor or nuclear signaling factor (15).

Epistasis in laboratory animals can be explored by inducing mutation by techniques such as gene editing and selective breeding. In humans, epistasis can only be determined if there is a family where the two or more mutations segregate with different family members. Ideally there should be an individual with wild type sequence of the genes as a control (15).

In other cases, the two mutations may lie on different signaling pathways and may not have a synergistic interaction. Each mutation contributes to the phenotype in an additive manner. A disease severity score can help determine if there is an epistatic interaction between the two mutations at a clinical level. Similarly, a test such as *in vitro* antibody production can also determine if there is epistasis at a biochemical level. The probability of epistasis can be assessed by an epistasis score (15). Epistasis occurs when the genetic, biochemical and clinical scores are congruent.

In another scenario, patients who have large deletions involving many immune system genes present difficulties in determining epistasis. Although the large deletion might be present in other family members, the individual genes do not segregate. In this case, the comparison is between a patient having the large deletion involving many genes with other unrelated patients having a single gene deletion. The interindividual phenotypic differences are likely to be much greater than those of a single kindred, where two or more genes segregate. This was seen in the CTLA4 Gene Complex Deletion Syndrome (CDCGS) where large deletions of chromosome 2 results in deletion of CTLA4, CD28 and ICOS (16, 17).

**Figure 1:**
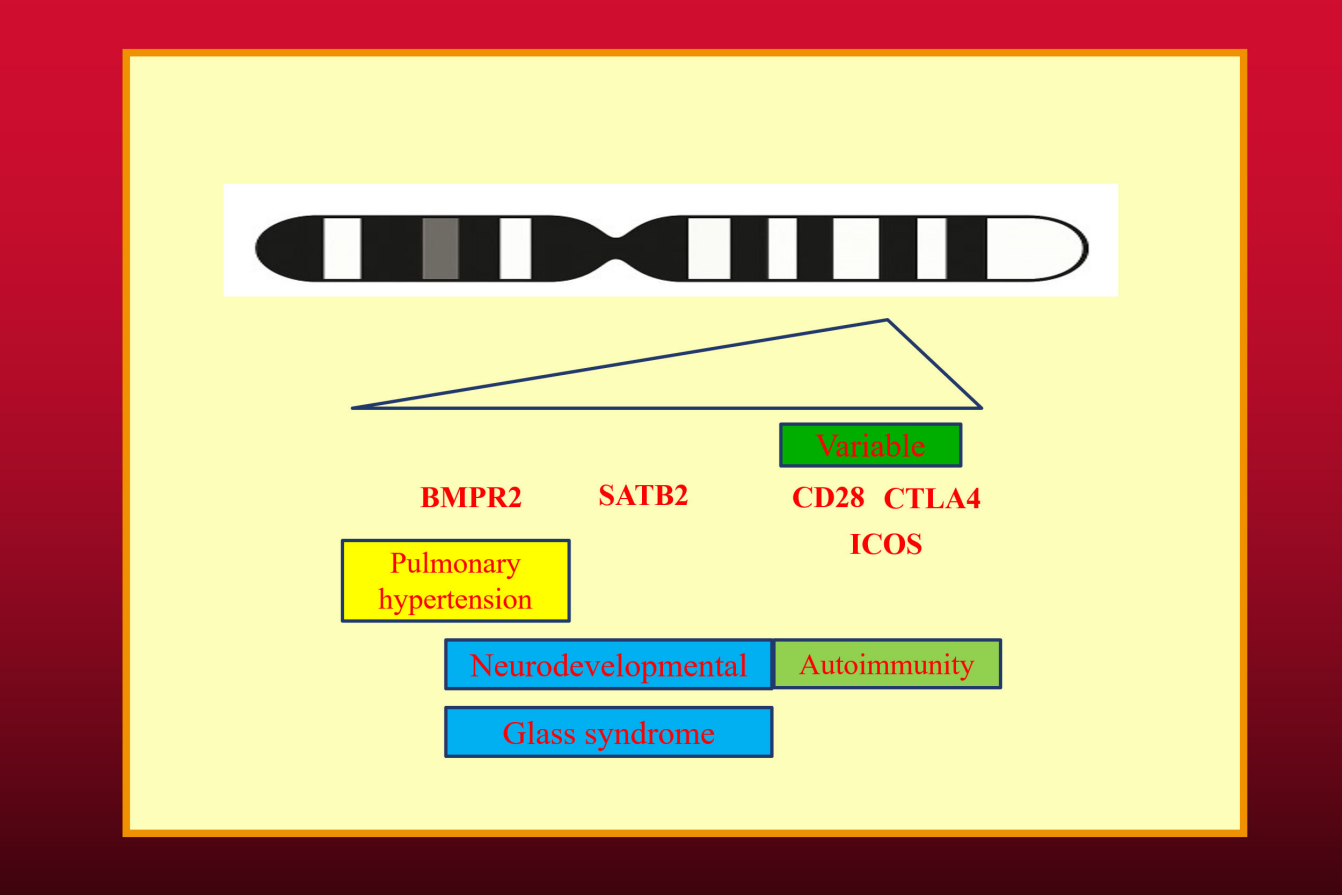
The CTLA4 Gene Cluster Deletion syndrome (CGCDS). The spectrum of phenotypic features vary from CTLA4 haploinsufficiency to Glass Syndrome and Pulmonary hypertension, depending on deleted genes. The banded chromosome was generated by Microsoft co-pilot.

In these large deletions, the loss of CTLA4 causes haploinsufficiency resulting in a CVID-like disorder with severe autoimmunity associated with hypogammaglobulinemia. ICOS deficiency is an autosomal recessive disorder also resulting in a CVID-like disorder. CD28 deficiency is another autosomal recessive condition leading to severe cutaneous HPV infection, which has been termed the “Tree man syndrome” (18). Some patients with CGCDS have deletion of SATB2 resulting in a severe neurodevelopmental delay (Glass syndrome) and BMPR2 leading to pulmonary hypertension (**Figure 1**) (17). There is thus substantial phenotypic variability of the CGCDS depending on the deleted genes (16).

In these kindreds, the heterozygous CTLA4 mutation appears to be the critical mutation resulting in severe autoimmunity. While it is clear this is a polygenic CVID-like disorder, the role of the heterozygous CD28 and ICOS deletions are uncertain. As noted above, epistasis cannot be determined in large deletions, as these mutations do not segregate in different family members.

## Conclusion

In conclusion, it is apparent that an increasing number of PIDs/IEIs are consequent to polygenic disorders. In polygenic disorders, each variant should be curated to determine its contribution to the phenotype. It is also apparent from the examples presented here that not all cases of digenic or higher order polygenic disorders are examples of epistasis. Given the increasing recognition of polygenic PIDs/IEIs, there should be a separate category for these disorders in the IUIS PID classification system.

## References

[1] Cunningham-Rundles C Bodian C. Common variable immunodeficiency: clinical and immunological features of 248 patients. Clin Immunol. (1999) 92:34–48. doi: 10.1006/clim.1999.4725, PMID: 10413651

[2] Ameratunga R Ahn Y Jordan A Lehnert K Brothers S Woon ST. Keeping it in the family: the case for considering late-onset combined immunodeficiency a subset of common variable immunodeficiency disorders. Expert Rev Clin Immunol. (2018) 14:549–56. doi: 10.1080/1744666X.2018.1481750, PMID: 29806948

[3] Abolhassani H Hammarstrom L Cunningham-Rundles C. Current genetic landscape in common variable immune deficiency. Blood. (2020) 135:656–67. doi: 10.1182/blood.2019000929, PMID: 31942606 PMC7046605

[4] Abolhassani H Chou J Bainter W Platt CD Tavassoli M Momen T. Clinical, immunologic, and genetic spectrum of 696 patients with combined immunodeficiency. J Allergy Clin Immunol. (2018) 141:1450–8. doi: 10.1016/j.jaci.2017.06.049, PMID: 28916186

[5] Ameratunga R Woon ST Gillis D Koopmans W Steele R. New diagnostic criteria for common variable immune deficiency (CVID), which may assist with decisions to treat with intravenous or subcutaneous immunoglobulin. Clin Exp Immunol. (2013) 174:203–11. doi: 10.1111/cei.12178, PMID: 23859429 PMC3828823

[6] Bonilla FA Barlan I Chapel H Costa-Carvalho T Cunningham-Rundles C Morena MT. International consensus document (ICON): common variable immunodeficiency disorders. J Allergy Clin Immunol Pract. (2016) 4:38–59. doi: 10.1016/j.jaip.2015.07.025, PMID: 26563668 PMC4869529

[7] Seidel MG Kindle G Gathmann B Quinti I Buckland M Montfrans van J. The european society for immunodeficiencies (ESID) registry working definitions for the clinical diagnosis of inborn errors of immunity. J Allergy Clin Immunol Pract. (2019) 7:1763–70. doi: 10.1016/j.jaip.2019.02.004, PMID: 30776527

[8] Tangye SG Al-Herz W Bousfiha A Cunningham-Rundles C Franco JL Holland SM. Human inborn errors of immunity: 2022 update on the classification from the international union of immunological societies expert committee. J Clin Immunol. (2022) 24:1–35. doi: 10.1007/s10875-022-01289-3, PMID: 35748970 PMC9244088

[9] Ameratunga R Edwards ESJ Lehnert K Lehnert K Leung E Woon S-T. The rapidly expanding genetic spectrum of Common Variable Immunodeficiency-like disorders. J Allergy Clin Immunol Pract. (2023) 14:00174–5. doi: 10.1016/j.jaip.2023.01.048, PMID: 36796510

[10] Richards S Aziz N Bale S Bick D Das S Gastier-Foster J. Standards and guidelines for the interpretation of sequence variants: a joint consensus recommendation of the American College of Medical Genetics and Genomics and the Association for Molecular Pathology. Genet Med. (2015) 17:405–24. doi: 10.1038/gim.2015.30, PMID: 25741868 PMC4544753

[11] Koopmans W Woon ST Brooks AE Dunbar PR Browett P Ameratunga R. Clinical variability of family members with the C104R mutation in transmembrane activator and calcium modulator and cyclophilin ligand interactor (TACI). J Clin Immunol. (2013) 33:68–73. doi: 10.1007/s10875-012-9793-x, PMID: 22983507

[12] Ameratunga R Koopmans W Woon ST Leung E Lehnert K Slade CA. Epistatic interactions between mutations of TACI (TNFRSF13B) and TCF3 result in a severe primary immunodeficiency disorder and systemic lupus erythematosus. Clin Transl Immunol. (2017) 6:e159. doi: 10.1038/cti.2017.41, PMID: 29114388 PMC5671988

[13] Ameratunga R. Assessing disease severity in common variable immunodeficiency disorders (CVID) and CVID-like disorders. Front Immunol. (2018) 9:2130. doi: 10.3389/fimmu.2018.02130, PMID: 30323807 PMC6172311

[14] Ameratunga R Longhurst H Lehnert K Steele R Edwards ESJ Woon ST. Are all primary immunodeficiency disorders inborn errors of immunity? Front Immunol. (2021) 12:706796.eCollection. doi: 10.3389/fimmu.2021.706796.eCollection 34367167 PMC8335567

[15] Ameratunga R Woon ST Bryant VL Steele R Slade C Leung EY. Clinical Implications of Digenic inheritiance and epistasis in Primary Immunodeficiency Disorders. Front Immunol. (2018) 8:1965. doi: 10.3389/fimmu.2017.01965, PMID: 29434582 PMC5790765

[16] Le Coz C Nolan BE Trofa M Kamsheh AM Khokha MK Lakhani SA. Cytotoxic T-lymphocyte-associated protein 4 haploinsufficiency-associated inflammation can occur independently of T-cell hyperproliferation. Front Immunol. (2018) 9:1715. doi: 10.3389/fimmu.2018.01715, PMID: 30087679 PMC6066513

[17] Brakta C Tabet AC Puel M Pacault M Stolzenberg M-C Goudet C. 2q33 deletions underlying syndromic and non-syndromic CTLA4 deficiency. J Clin Immunol. (2024) 45:46. doi: 10.1007/s10875-024-01831-5, PMID: 39578275

[18] Béziat V Rapaport F Hu J Titeux M Claustres des Bonnet M Bourgey M. Humans with inherited T cell CD28 deficiency are susceptible to skin papillomaviruses but are otherwise healthy. Cell. (2021) 184:3812–28.e30., PMID: 34214472 10.1016/j.cell.2021.06.004 PMC8329841

